# Pleural Metastasis as an Initial Presentation of Prostate Cancer: Case Report and Literature Review

**DOI:** 10.3390/diagnostics15060666

**Published:** 2025-03-10

**Authors:** Katarzyna Skrobisz, Kevin Miszewski, Laura Miszewska, Michał Bieńkowski, Marcin Matuszewski, Michał Studniarek

**Affiliations:** 1 Department of Radiology, Medical University of Gdańsk, 17 Smoluchowskiego St., 80-214 Gdańsk, Poland; kskrobisz@gumed.edu.pl (K.S.);; 2 Department of Urology, Medical University of Gdańsk, 17 Smoluchowskiego St., 80-214 Gdańsk, Poland; 3 Faculty of Medicine, Medical University of Gdańsk, 17 Smoluchowskiego St., 80-214 Gdańsk, Poland; 4 Department of Pathology, Medical University of Gdańsk, 17 Smoluchowskiego St., 80-214 Gdańsk, Poland

**Keywords:** prostate cancer, pleural metastasis, low PSA, metastatic disease

## Abstract

**Background and Clinical Significance**: Prostate cancer (PCa) is among the most commonly diagnosed malignancies in men worldwide. While bone and lymph nodes are the most frequent metastatic sites, prostate cancer cells have the potential to spread to virtually any organ, including the pleura, which is an exceedingly rare initial site of presentation that can mimic mesothelioma or primary lung cancer. **Case Presentation**: We describe a 77-year-old man who presented with exertional dyspnea and intermittent cough, initially suggesting a cardiopulmonary etiology. Imaging revealed multiple pleural nodules and an extensive right-sided pleural effusion. Despite a borderline serum prostate-specific antigen (PSA) level of 2.91 ng/mL, histopathology and immunohistochemistry of pleural biopsies confirmed metastatic prostate adenocarcinoma. Subsequent imaging identified a PIRADS 5 lesion in the prostate, and a biopsy confirmed ISUP Grade Group 5 disease (Gleason score 4 + 5 = 9). A bone scan showed no skeletal metastases, and a contrast-enhanced CT of the abdomen found no additional metastatic lesions. The patient was started on androgen deprivation therapy followed by abiraterone. This case underscores the diagnostic challenge posed by atypical metastatic presentations of prostate cancer. Low or moderately elevated PSA can obscure suspicion of prostate origin, especially with pleural-based lesions suggestive of mesothelioma. Immunohistochemical markers, including androgen receptors, AMACR, and Prostein, are critical for accurate diagnosis. **Conclusions**: Clinicians must maintain a high index of suspicion for prostate cancer in older men with unexplained pleural effusions, nodules, or masses, even with low-normal PSA levels. Early recognition and prompt treatment can improve outcomes, despite the rarity and aggressiveness of pleural metastases.

## 1. Introduction

Prostate cancer (PCa) remains one of the most commonly diagnosed malignancies in men worldwide and a leading cause of cancer-related mortality [1,2]. The metastatic potential of prostate cancer is broad, typically involving bone and regional lymph nodes. However, in rare circumstances, the pleura can be a site of metastatic spread. Clinically evident pleural metastases from PCa, particularly when presenting as the first manifestation of disease, are exceedingly uncommon. Indeed, multiple large autopsy series have shown that although up to 20–46% of patients with advanced prostate cancer may have some degree of pulmonary or pleural involvement on post-mortem evaluation, only a small subset manifest clinically significant pleural effusions or pleural-based lesions during lifetime [3,4,5,6,7].

While pleural metastases from PCa are frequently overshadowed by the far more common skeletal and lymphatic spread, its clinical presentation can be highly varied: from small, asymptomatic pleural nodules discovered incidentally, to rapidly re-accumulating or massive hemorrhagic effusions that precipitate dyspnea, cough, or chest discomfort [6,7,8,9]. Moreover, pleural-based lesions from PCa can mimic primary pleural tumors (e.g., mesothelioma) or metastatic disease originating from more common primary sites in men, such as the lung, colorectal, or kidney [10]. Low or modestly elevated prostate-specific antigen (PSA) levels can further obscure clinical suspicion of a prostatic origin [11,12,13,14]. Consequently, immunohistochemical (IHC) staining for PSA, prostate acid phosphatase, NKX 3.1, androgen receptor (AR), Prostein, and/or alpha-methylacyl-CoA racemase (AMACR) remains pivotal in establishing the diagnosis [8,10,15].

Given its rarity and diagnostic challenges, pleural metastasis from PCa warrants vigilant assessment. Herein, we describe a case of a 77-year-old man with pleural effusion and nodular pleural thickening as the initial presentation of PCa. We integrate this case with a comprehensive literature review on pleural involvement in prostate cancer, highlight the relevant diagnostic workup, and emphasize the importance of recognizing this infrequent but significant pattern of spread.

## 2. Case Presentation

**Figure 1:**
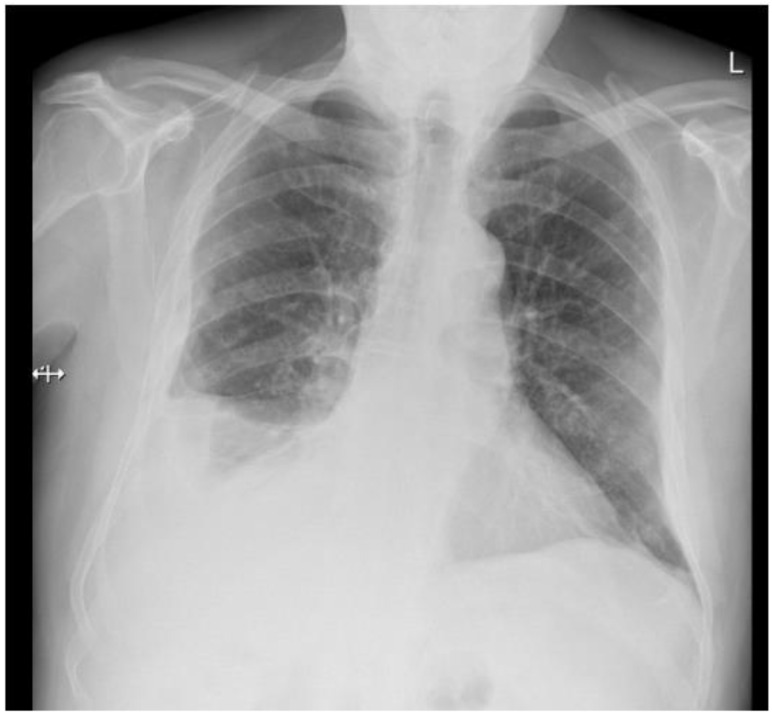
Chest X-ray—right pleural effusion, thickening of the pleura on the right side, bilateral small nodules.

**Figure 2:**
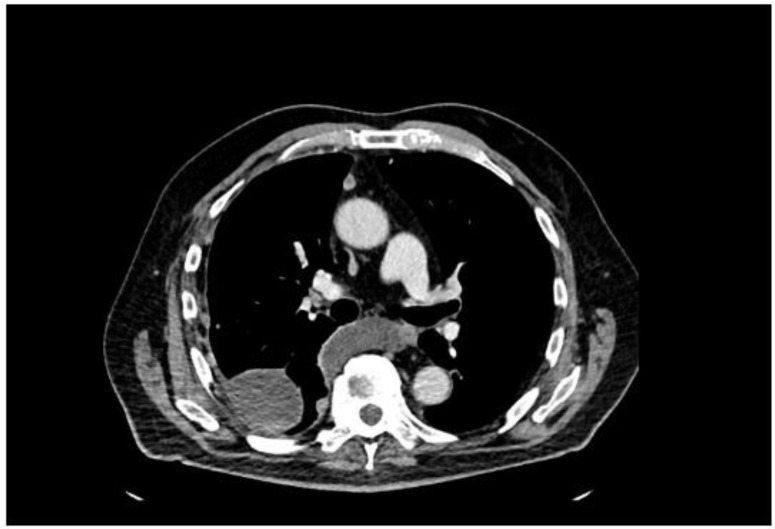
CT—axial plane, after contrast administration, right-sided effusion, small enhancing after contrast administration pleural nodules.

**Figure 3:**
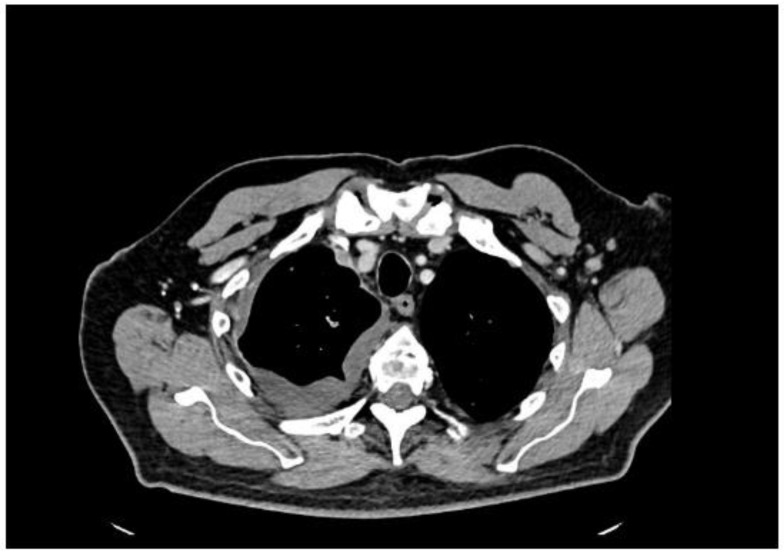
CT—axial plane, after contrast administration, right-sided encysted pleural fluid and small enhancing after contrast administration pleural nodules.

A 77-year-old man presented with several weeks of exertional dyspnea and intermittent dry cough. His past medical history was notable for ankylosing spondylitis, chronic gastritis, a left salivary gland tumor resected 11 years prior (pathologic details unknown), and a cholecystectomy. He had a significant smoking history (20 pack-years) but quit 32 years earlier. An initial chest radiograph, obtained to evaluate possible heart failure, revealed a large right-sided pleural effusion. Figure 1 A contrast-enhanced computed tomography (CT) of the chest further delineated multiple pleural nodules (up to 17 mm) and diffuse, bilateral small pulmonary nodules. Figure 2 and Figure 3 Two thoracenteses drained ~3800 mL of hemorrhagic pleural fluid in total. Fluid cytology was negative for malignancy, and no cell block preparation was performed. Further evaluation with bronchoscopy and bronchoalveolar lavage was unremarkable for infection or malignant cells.

**Figure 4:**
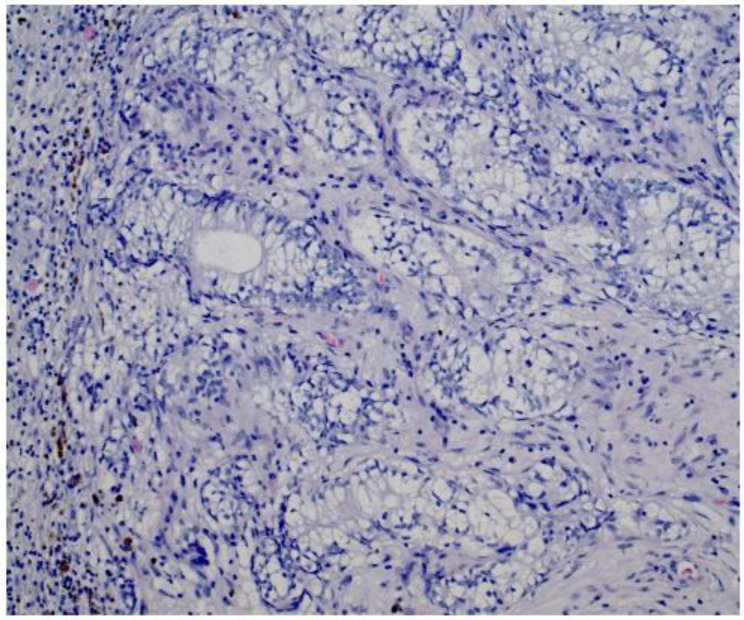
HE image showing pleural fibrous tissue infiltrated by clear cell adenocarcinoma (positive for panCK, AR and AMACR, negative for TTF1, not shown). Magnification: 20×.

**Figure 5:**
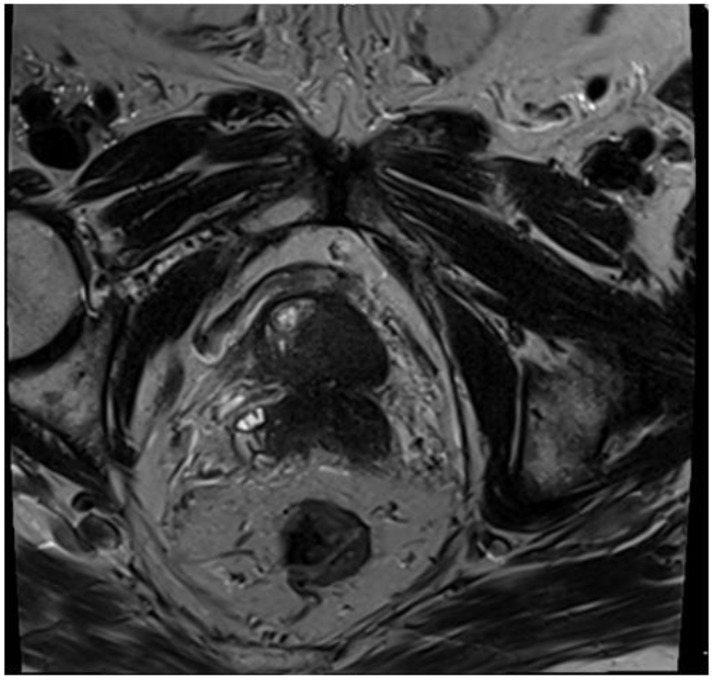
MR T2 weighted-images—axial plane, extensive infiltration of the gland with involvement of the seminal vesicles—T3b.

**Figure 6:**
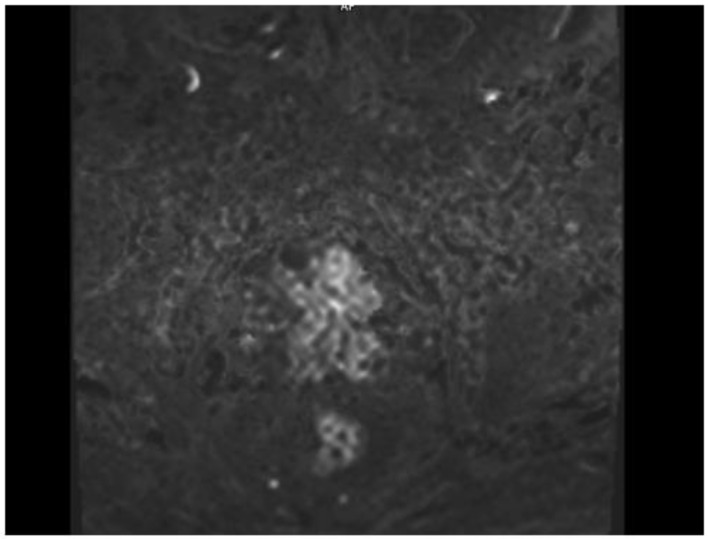
MR—diffusion-weighted imaging 2000, axial plane, hyperintense lesion indicating restricted diffusion is observed.

**Figure 7:**
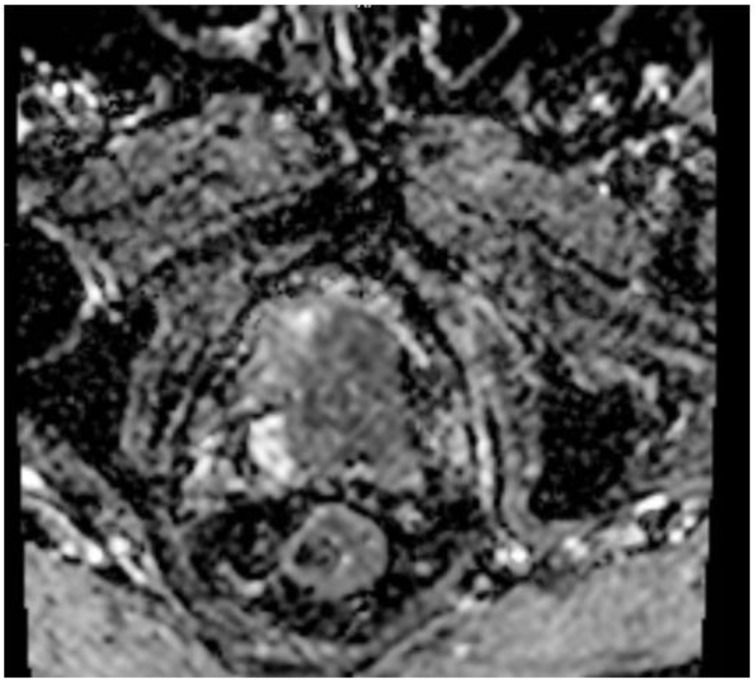
MR—ADC map. On the corresponding ADC map, the lesion appears hypointense.

**Figure 8:**
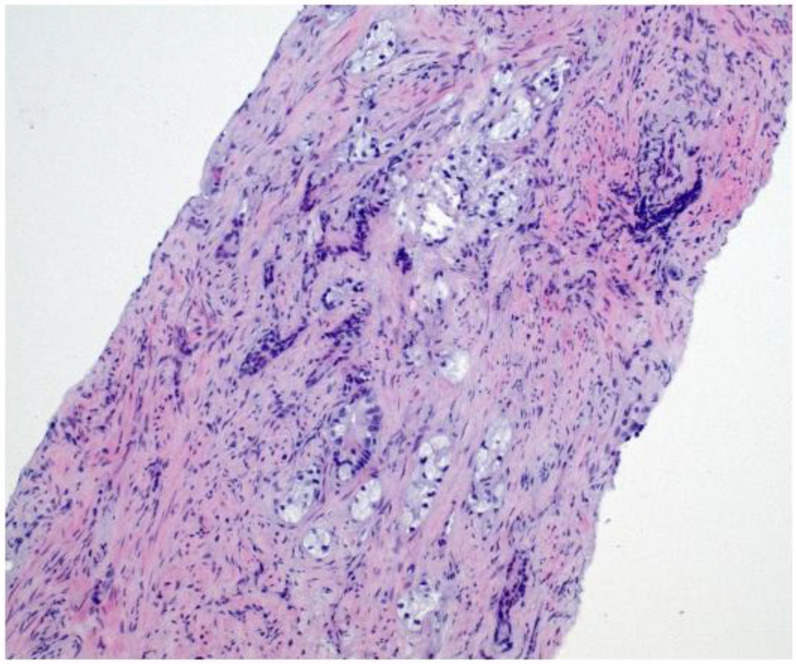
HE image of prostatic core needle biopsy showing the infiltration of adenocarcinoma with poorly formed glands (Gleason pattern 4) and clear cytoplasm. Magnification: 10×.

Given the persistent re-accumulation of pleural effusion and suspicious nodular pleural thickening, a diagnostic video-assisted thoracoscopic surgery (VATS) was performed. Approximately 900 mL of bloody fluid was drained, and multiple pleural nodules were biopsied. Histopathology revealed metastatic carcinoma. Immunohistochemical staining demonstrated positivity for androgen receptor (AR), alpha-methylacyl-CoA racemase (AMACR), and focal Prostein, alongside negativity for TTF1, CK20, WT1, D2-40, PAX8, GATA3, and CDX2, consistent with a primary prostatic origin [8]. Figure 4 Digital rectal examination (DRE) revealed a firm prostate gland with bilateral palpable nodules consistent with clinical stage T2c disease. Although the patient’s serum PSA was only 2.91 ng/mL, a subsequent prostate MRI revealed a PIRADS 5 lesion invading bilateral seminal vesicles Figure 5, Figure 6 and Figure 7, and a transrectal fusion biopsy confirmed ISUP grade group five disease (Gleason 4 + 5 = 9). Figure 8 A bone scan showed no skeletal metastases, and a contrast-enhanced CT of the abdomen found no evidence of abdominal metastatic disease (notable findings included multiple simple hepatic and renal cysts, and a homogeneous left adrenal adenoma). The patient was staged as cT3bN0M1 (pleural metastasis) and started on androgen deprivation therapy with bicalutamide plus leuprolide, followed shortly by abiraterone and low-dose corticosteroids. Three-month follow-up imaging demonstrated stable pleural disease and partial symptomatic relief in his respiratory complaints.

## 3. Literature Review and Discussion

### 3.1. Overview of Prostate Cancer Metastatic Patterns

Prostate cancer’s metastatic propensity predominantly targets the skeletal system (especially the axial skeleton) and regional lymph nodes, yet atypical sites including the lungs, liver, adrenal glands, and pleura are described [1,2,3,6]. Bone involvement may be seen in as many as 90% of patients with advanced PCa at autopsy, typically in osteoblastic form [4,16]. Less frequently, visceral metastases—particularly isolated or solitary ones—can occur in the liver (~25–30%), lungs (~20–46% on autopsy), and pleura (~1–5% clinically, but up to ~21% on autopsy) [3,5,7]. Adrenal glands are also involved in a subset of advanced cases (17–20% at autopsy), though these lesions may remain asymptomatic and undetected during life unless they enlarge or cause hormonal imbalances [4]. Less frequently, prostate cancer spreads to the brain, with <1% of patients developing central nervous system lesions. When brain metastases do occur, they typically reflect end-stage disease, and survival after detection is limited [17]. Metastatic deposits to the male breast tissue, while exceedingly rare, have also been reported, occasionally in the context of estrogen-based therapies that may predispose to breast tissue changes [18,19,20,21]. Another uncommon phenomenon is ocular metastasis. Although ocular metastases (mainly to the choroid or orbit) are well-recognized in other malignancies, prostate involvement is highly uncommon. In fact, ocular or orbital metastases typically occur in about 5–10% of metastatic cases, and they generally present as part of a more extensive metastatic process rather than as an isolated initial manifestation [22,23]. Finally, isolated case or autopsy reports have documented metastases to the kidneys, pancreas, muscles, spleen, testes, and penis, emphasizing the versatility of advanced PCa to colonize nearly any organ [5,24].

Despite up to one-fifth of autopsy cases exhibiting pleural involvement, clinically evident pleural effusion or nodular pleural metastasis from PCa remains relatively rare. For instance, a retrospective series by Vinjamoori et al. [3] identified only 33 of 620 (5%) patients with atypical lung or pleural metastases over a 10-year period. In that study, purely pleural-based disease (effusions, nodules, or masses) was found in <1% of patients. Another radiologic analysis reported ~22% of advanced PCa patients with pleural fluid, only 13.6% of which was malignant upon cytologic evaluation [8].

A thorough search of the literature reveals less than 20 documented cases of clinically recognized pleural metastases from PCa, many reported as single-patient case studies [6,9,13,14,25,26,27,28,29,30,31,32,33]. Depending on the inclusion criteria (e.g., autopsy-only data versus antemortem diagnoses, the presence of concurrent lung parenchymal metastases, or the presence of an isolated pleural effusion), the exact count varies slightly. Nevertheless, all sources agree that symptomatic pleural metastases from PCa are distinctly uncommon. In older literature, Long and Husband [6] highlighted that out of 398 patients with prostate cancer undergoing abdominal CT, adrenal metastasis was found in only one case, and no mention of malignant pleural disease was made, reinforcing the rarity of clinically evident pleural lesions.

Among the published accounts, some patients present with massive pleural effusions, recurrent hemorrhagic effusions, or nodular pleural masses that mimic mesothelioma or advanced lung cancer [9,13,26,27,34,35]. Others demonstrate unusual transudative effusions or extrathoracic spread that confounds diagnosis [36]. In many such reports, serum PSA levels varied widely; some patients had extremely high PSA, whereas others, paradoxically, maintained near-normal or mildly elevated levels [14,37]. The capacity of advanced, high-grade, or neuroendocrine-dedifferentiated prostate cancer to secrete minimal PSA is well documented, adding another layer of diagnostic complexity [11,12,14,38].

### 3.2. Clinical Features and Diagnostic Challenges

Patients with pleural-based metastatic disease may present with nonspecific respiratory symptoms—dyspnea, cough, chest discomfort—and may or may not show features suggestive of a prostatic origin [9,25]. A borderline or even normal PSA can substantially diminish clinical suspicion of PCa. Therefore, a systematic approach to new-onset pleural effusions in older men should maintain a differential diagnosis that includes metastatic prostate cancer, especially if other etiologies (e.g., primary lung malignancy, mesothelioma, or tuberculosis) are not confirmed.

The gold standard for diagnosing malignant pleural effusion is cytologic detection of tumor cells in the fluid or tissue confirmation from pleural biopsies. However, pleural fluid cytology can be falsely negative, particularly for prostate cancer. Some studies cite detection rates as low as 40–50% in malignant pleural effusions overall (all tumor types), and possibly even lower in PCa [8,39]. Repeated thoracenteses might marginally improve yields, but the most definitive diagnostic approach often involves pleural biopsy, either via VATS or medical thoracoscopy [6,9,14,27,35]. In the setting of negative fluid cytology, tissue sampling can clarify the diagnosis.

### 3.3. Role of Immunohistochemistry

Given the nonspecific morphological features of metastatic adenocarcinoma, IHC staining is indispensable when PCa is suspected. The typical prostatic markers include PSA, prostate-specific acid phosphatase, NKX 3.1, and AMACR (P504S). Additional or alternative markers, such as Prostein (P501S) and androgen receptor (AR), may also be employed [8,10,15]. Of these, PSA is the most used clinical screening and monitoring test, yet pleural fluid PSA alone—when measured—can also be diagnostic if high. In many documented cases, pleural fluid PSA was significantly elevated (in the range of hundreds or thousands ng/mL), though exceptions exist [9,10,37]. Meanwhile, negative or low IHC positivity for TTF1, CK20, or mesothelioma markers (e.g., calretinin, WT1, D2-40) helps exclude lung or mesothelial primary tumors.

### 3.4. Therapeutic and Prognostic Considerations

Pleural disease typically indicates advanced or high-grade prostate cancer and often represents an aggressive tumor subtype [1,5,8,40]. While hormone-sensitive disease may still respond to androgen deprivation therapy (ADT) with GnRH analogs/antagonists ± anti-androgens, the addition of abiraterone acetate or novel anti-androgens (e.g., enzalutamide, apalutamide) can improve survival in high-volume metastatic disease [41]. Taxane-based chemotherapy (e.g., docetaxel) is often employed in castration-resistant or rapidly progressing disease [42]. Pleural involvement may manifest as malignant pleural effusions, requiring symptomatic management such as thoracenteses, indwelling pleural catheters, or pleurodesis. Although the overall prognosis with pleural metastases is guarded, timely systemic therapy can extend survival and relieve symptoms [9,14].

In our case, the patient achieved partial symptom control and stable radiographic findings following ADT (leuprolide plus bicalutamide) with early intensification using abiraterone. Notably, his PSA was only borderline elevated despite extensive pleural involvement. The pivotal diagnostic step was a VATS-based pleural biopsy with immunohistochemical confirmation. While pleural metastases generally imply advanced disease, outcomes vary. Some patients respond well to combined androgen blockade or newer androgen signaling inhibitors, whereas others progress more rapidly due to high-grade or neuroendocrine features [11,16,38]. Maintaining quality of life by controlling local symptoms (e.g., repeated effusion drainage) and employing combination therapies remains paramount [40]. In hormone-refractory or castration-resistant cases with pleural involvement, salvage chemotherapy and supportive interventions are considerations. Early, accurate diagnosis is therefore critical to guide appropriate systemic treatment.

### 3.5. Composite Review of Published Cases

Prostate cancer rarely metastasizes to the pleura, and only a handful of clinical cases, approximately five [9,14,25,26,27,33], have been documented in the medical literature to this date. We have summarized clinical observations in Table 1, focusing on their key details, diagnostic imaging, PSA patterns, and immunohistochemical findings. Although bone and lymph node involvement predominate in advanced prostate cancer [4,6] pleural metastases—especially those manifesting with massive, recurrent, or hemorrhagic effusions—have been increasingly identified. Interestingly, many such cases arose in patients with high Gleason scores or castration-resistant disease; yet exceptions exist in which PSA levels remained within near-normal ranges [9,14]. Radiologic appearances also varied, including solitary pleural nodules, diffuse nodular pleural thickening, and effusions that can mimic pneumonia, mesothelioma, or primary lung cancer [14,33]. The published accounts underscore that definitive diagnosis typically hinges on pleural biopsy and immunostaining for prostate-specific markers (for example, PSA and NKX 3.1), given the low yield of pleural fluid cytology alone [9,26]. Recognizing this atypical metastatic pattern is crucial for timely therapy and potentially improved symptom control.

Kawahara et al. [25] presented a 65-year-old man whose bilateral adrenal gland involvement preceded the discovery of pleural metastases. This patient had moderately differentiated prostate adenocarcinoma; after a period of therapy, imaging revealed pleural lesions alongside rising prostate-specific antigen (PSA) levels. Notably, the investigators cited autopsy data suggesting that while around 20% of advanced prostate cancer cases may exhibit adrenal involvement, pleural metastasis can also be detected in a similar percentage at autopsy-yet less frequently recognized clinically. This patient developed the pleural metastasis after docetaxel therapy had seemingly lost effectiveness, highlighting how hormonally resistant disease can spread to less common sites.

Another interesting case was described by Yasuda et al. [9], involving a 71-year-old man who initially had lung metastases from prostate cancer that responded to hormonal therapy, but who later developed a solitary pleural thickening. Imaging alone did not clearly distinguish this lesion from other pleural processes. Ultimately, pathological evaluation demonstrated prostate adenocarcinoma cells positive for PSA. The authors underscored how a minimal rise in serum PSA does not exclude new metastatic sites, and how a pleural metastasis can appear after lung lesions have regressed.

Vakil et al. [14] reported a patient with previously treated prostate cancer who presented with a large pleural effusion despite having normal PSA levels. Pleural fluid cytology revealed an adenocarcinoma, but the absence of an elevated PSA, either in serum or fluid, initially complicated the diagnosis. Additional immunohistochemistry showed positivity for chromogranin A and carcinoembryonic antigen, helping conclude that this was a neuroendocrine-like variant of prostate cancer metastasis. As the authors emphasized, some prostate tumors can dedifferentiate and secrete negligible PSA, which can mask the link to the prostate origin.

A more dramatic presentation was seen in the report by dos Santos et al. [26], where a 72-year-old man arrived with massive right-sided pleural effusion, fever, and weight loss. He had previously undergone prostatectomy, radiation, orchiectomy, and docetaxel-based chemotherapy. Despite repeated thoracenteses, initial cytological samples did not confirm malignancy. Eventually, imaging suggested pleural nodules, and a biopsy confirmed metastatic prostate adenocarcinoma which was immunopositive for PSA. The authors stressed how fluid cytology alone can be misleading, particularly if the tumor cells are scant or morphologically subtle. In that scenario, pleural biopsy and immunohistochemical stains remain essential to secure the diagnosis.

Sampsonas et al. [27] described a 91-year-old patient who presented with large unilateral pleural effusion, shortness of breath, and no prior prostate cancer history. Thoracic imaging revealed mediastinal pleural thickening, but the key diagnostic step was endobronchial ultrasound-guided biopsy (EBUS-TBNA). Histopathology showed malignant cells positive for PSA and alpha-methylacyl-CoA racemase (AMACR), confirming a primary prostatic origin. This underscores how advanced sampling techniques can definitively identify pleural disease caused by prostate cancer, even in the absence of known disease elsewhere. The authors noted that the patient’s serum PSA was moderately elevated (135 ng/mL), but not as high as might be expected for widely metastatic disease.

Singh et al. [33] offered another instructive example: a 70-year-old man with a history of prostate adenocarcinoma who developed recurrent right-sided pleural effusions that were repeatedly negative on fluid cytology. Initially, the effusion was mistakenly attributed to pneumonia or possible lung cancer. Only after VATS pleurodesis was performed, motivated by the patient’s rapidly re-accumulating fluid, were multiple metastatic nodules seen on the pleural surface. Biopsy with immunohistochemistry confirmed metastatic prostate cancer cells. This case highlights how repeated negative thoracentesis results do not rule out malignancy, especially for prostate carcinoma, which may have low cellularity in effusions. Ultimately, pleural biopsy is often necessary if suspicion remains.

Collectively, these published cases highlight several important themes. First, the PSA level can be unreliable or misleading. While many patients with pleural metastasis have significantly elevated PSA, a substantial subgroup show only modest or even normal PSA levels [13,14]. This phenomenon may stem from either dedifferentiation or neuroendocrine differentiation of the tumor, leading to diminished PSA production. Second, standard cytologic evaluation of pleural fluid may fail to detect malignant prostatic cells, resulting in repeated non-diagnostic tests [26,33]. Pleural biopsies via VATS or medical thoracoscopy are often decisive. Third, immunohistochemical markers such as PSA, prostate-specific acid phosphatase (PSAP), NKX 3.1, AMACR (P504S), Prostein (P501S), or androgen receptor (AR) are typically used to confirm prostatic origin. When these markers are positive, especially in conjunction with negative stains for TTF1 or mesothelial markers (e.g., calretinin), a prostatic source is generally established [9,10].

Regarding treatment, the presence of pleural disease often indicates advanced prostate cancer requiring aggressive systemic therapy [3,4]. Patients may receive androgen deprivation therapy (GnRH analogs or antagonists plus anti-androgens), and in hormone-refractory situations, additional chemotherapy or new-generation androgen receptor inhibitors (abiraterone, enzalutamide) might be initiated. The management of pleural effusions themselves can involve repeated thoracenteses, chest tube placement, indwelling pleural catheters, or pleurodesis to improve respiratory symptoms. Some patients, like in Kawahara’s case [25], progress rapidly if the disease is highly resistant, while others achieve partial control for several months or years [27]. Overall, these publications illustrate the crucial role of imaging, repeated fluid analyses, and especially pleural biopsy with extended IHC in clarifying ambiguous or atypical thoracic findings in PCa patients.

### 3.6. Diagnostic Pearls

- Maintain High Index of Suspicion: In older males with unexplained pleural effusion, especially hemorrhagic or exudative in nature, consider prostate cancer even if PSA is normal or borderline;
- Repeat Thoracenteses: If cytology is negative yet clinical suspicion remains high, repeated fluid sampling may yield malignant cells, but the yield can be limited;
- Pleural Biopsy: Thoracoscopic or image-guided biopsy is the most definitive step when fluid analyses are inconclusive, providing tissue for histopathology and IH;
- Immunohistochemical Analysis: Demonstration of PSA, NKX 3.1, AR, Prostein, or AMACR positivity, coupled with negative TTF1, WT1, GATA3, or CK20, is diagnostic for metastatic prostate adenocarcinoma in pleural tissue;
- Early Systemic Therapy: Prompt initiation of hormonal or combined therapies can significantly improve symptom control and potentially prolong survival.

## 4. Conclusions

Pleural metastasis as an initial or early manifestation of prostate cancer is exceedingly rare, with only a small number of cases reported in the literature. Our patient’s borderline PSA levels and repeatedly negative thoracenteses highlight how such atypical presentations can obscure a prostatic origin. Based on this finding and supporting literature, clinicians should remain vigilant for metastatic prostate cancer in older men presenting with unexplained pleural effusions—even in the absence of classic risk factors—since advanced or high-grade disease may secrete minimal PSA. Timely diagnosis via pleural biopsy and immunohistochemistry, followed by appropriate systemic therapy (e.g., androgen deprivation and next-generation hormonal agents), can help stabilize disease, palliate symptoms, and potentially improve quality of life.

## Figures and Tables

**Table 1:** Reported cases of pleural metastases from prostate cancer, highlighting initial presentation, diagnostic challenges, treatments, and outcomes.

Study (Year)	Initial Characteristics (Pleural Metastasis Discovery)	Age (Years)	Symptoms	Pathology/Gleason Score	Presence of Pleural Effusion	Changes in Cytology	Initial PSA (ng/mL)	Tumor Type	Surgery/Prostate Primary Treatment	Radiation Therapy	Other Treatments for mPCa	Stage or Status	Treatment for mPCa (at Pleural Metastasis)	Outcome and Conclusions
Kawahara et al. (2009) [25]	Bilateral adrenal metastases discovered first; pleural tumor noted after 8–12 cycles of docetaxel	65	Not stated	Gleason 4 + 3 = 7	Yes	Not stated	515	Adenocarcinoma	Patient received MAB therapy	Not stated	MAB then docetaxel	Advanced/metastatic	Continued docetaxel-based chemotherapy + steroid	Progression to bilateral adrenal and pleural metastases; case underscores rarity of bilateral adrenal + pleural spread
Yasuda et al. (2013) [9]	Solitary pleural thickening on imaging 2.5 years after multi-pulmonary metastases	71	Shortness of breath	Not stated	Yes	NA	187.1	Adenocarcinoma	Androgen-deprivation therapy	Not stated	Hormone therapy	Advanced/metastatic	Anti-androgen therapy continued	Pleural biopsy positive for prostate origin (PSA+). Highlights that pleural thickening without typical effusion can still be metastatic prostate cancer
Vakil et al. (2014) [14]	Large left-sided effusion in recurrent metastatic prostate cancer 6 years post radical prostatectomy	73	Pleuritic chest pain, dyspnea	Poorly differentiated Ca	Yes	Negative for PSA stain in pleural fluid; cells were positive for CEA and chromogranin	Normal at recurrence	Adenocarcinoma with neuroendocrine features	Radical prostatectomy (6 years prior)	NA	Hormonal therapy	Recurrent metastatic PCa	Anti-androgen therapy continued	Emphasizes that pleural fluid PSA can be normal or negative; diagnosis confirmed by immunohistochemical stains (CEA+, Chromogranin A+, negative pleural fluid PSA)
dos Santos et al. (2011) [26]	Massive right pleural effusion discovered with weight loss, respiratory symptoms; previous failure of docetaxel-based therapy	72	Fever, weight loss, cough, dyspnea	Gleason 6 (3 + 3)	Yes	Fluid cytology negative multiple times; biopsy with PSA+ tumor cells	485	Adenocarcinoma	Radical prostatectomy + bilateral orchiectomy + anti-androgens, plus docetaxel chemo	External beam RT	Continued docetaxel and supportive therapy (pleurodesis)	Castration-resistant	Pleural drainage and pleurodesis; continued systemic therapy	Pleural biopsy and immunohistochemistry (PSA+, CK7+) established pleural mets. Despite prior therapies, patient progressed with new effusion
Sampsonas et al. (2024) [27]	Large unilateral effusion and mediastinal pleural thickening found on CT	91	Progressive dyspnea	Not stated	Yes	Biopsy-proven (EBUS TBNA) positive for PSA, AMACR, negative for TTF-1	135	Adenocarcinoma	Not stated	Not stated	Not stated	Not stated	Androgen deprivation therapy	First known case in literature of diagnosing a prostate-cancer pleural metastasis specifically by EBUS-TBNA. Emphasizes the importance of sampling the pleura in atypical presentations
Singh et al. (2013) [33]	Persistent effusion misdiagnosed initially as pneumonia	70	Shortness of breath, cough	Not stated	Yes	Repeated negative cytology; final diagnosis via VATS (PSA+)	Not stated	Adenocarcinoma	Surgery + radiation therapy (5 yrs prior)	Yes	Leuprolide for recurrence; planned chemo and bisphosphonates	Hormone refractory	Ultimately chemo, bisphosphonates, VATS pleurodesis	Repeatedly negative fluid cytology despite visible pleural nodules; confirmed via PSA+ biopsy. Highlights that thoracoscopy can be crucial for diagnosis

## Data Availability

The original contributions presented in this study are included in the article. Further inquiries can be directed to the corresponding author.
